# PIT study: research into the Protocol Imaginary execuTion of self-injury

**DOI:** 10.1192/j.eurpsy.2022.1708

**Published:** 2022-09-01

**Authors:** M. Van Kampen, N. Kool, M. Deen

**Affiliations:** Parnassia Groep, Centrum Intensieve Behandeling, Den Haag, Netherlands

**Keywords:** EMDR ’add’, treatment ’add’, self-injury ’add’, addiction ’add’

## Abstract

**Introduction:**

Self-injury, defined as inflicting damage or pain to one’s own body, is a way to deal with unbearable emotions. Unfortunately, it can become addictive through the rewarding effect, and this makes it also really hard to stop with this destructive behavior. Currently, there is a lack of specific treatments.

**Objectives:**

The aim of the research is to investigate whether the “Protocol imaginary execution of self-damaging behavior” leads to a reduction of self-damaging behavior and the urge to self-damaging behavior.

**Methods:**

We have investigated the “Protocol Imaginary execuTion of self-injury” as a potential treatment for self-injury. In this protocol the patient is asked to imagine he/she is performing the self-injury and at the same time a distracting task is offered. This ensures the working memory is double burdened as is with EMDR. We expected a reduction of patient’s self-injurious behavior. For this study, a single-case experimental design with 11 clinical patients is used, aimed to investigate whether there is a functional relationship between the treatment, the urge to self-injure and the frequency and seriousness of the self-injury. Data are analyzed with a multivariate analysis. The results of this study will contribute to expanding and improving treatment options for self-injury.

**Results:**

At the moment the results are not yet available, but they will be known in April 2022.

**Conclusions:**

Respondents indicate that they experience more control over self-injurious behaviour. We hope to have confirmed this in April 2022 with the analyzed data.

**Disclosure:**

No significant relationships.

